# Supplementary figures and images for: Photonic bandgap properties of hyperuniform systems self-assembled in a microfluidic channel
Source: Sci Rep. 2026 May 23;16:23577. doi: 10.1038/s41598-026-36235-5 (PMC13421525; doi:10.1038/s41598-026-36235-5)

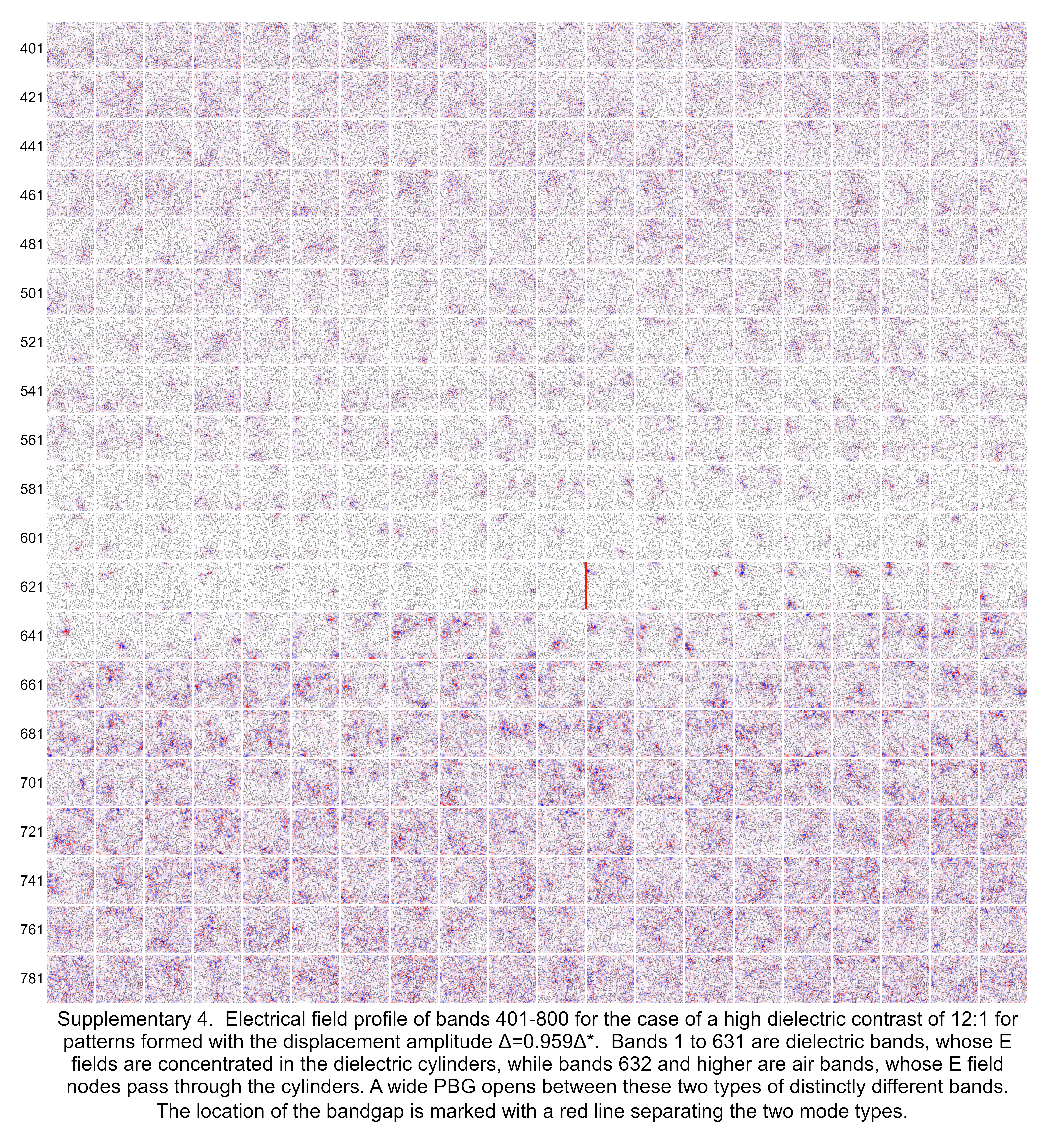

Supplement: Supplementary file 1 — Supplementary Material 1 [file 41598_2026_36235_MOESM1_ESM.jpg]

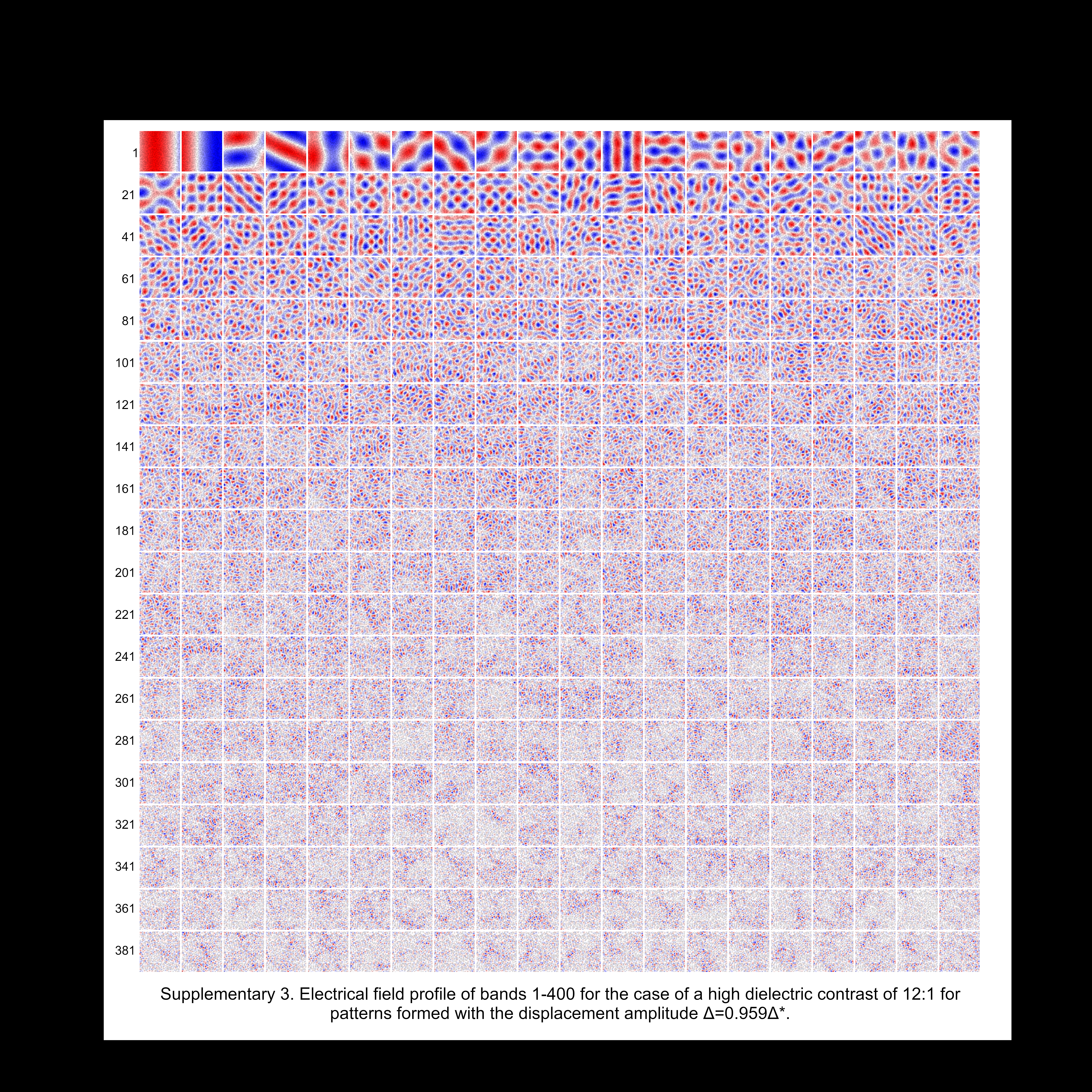

Supplement: Supplementary file 2 — Supplementary Material 2 [file 41598_2026_36235_MOESM2_ESM.jpg]

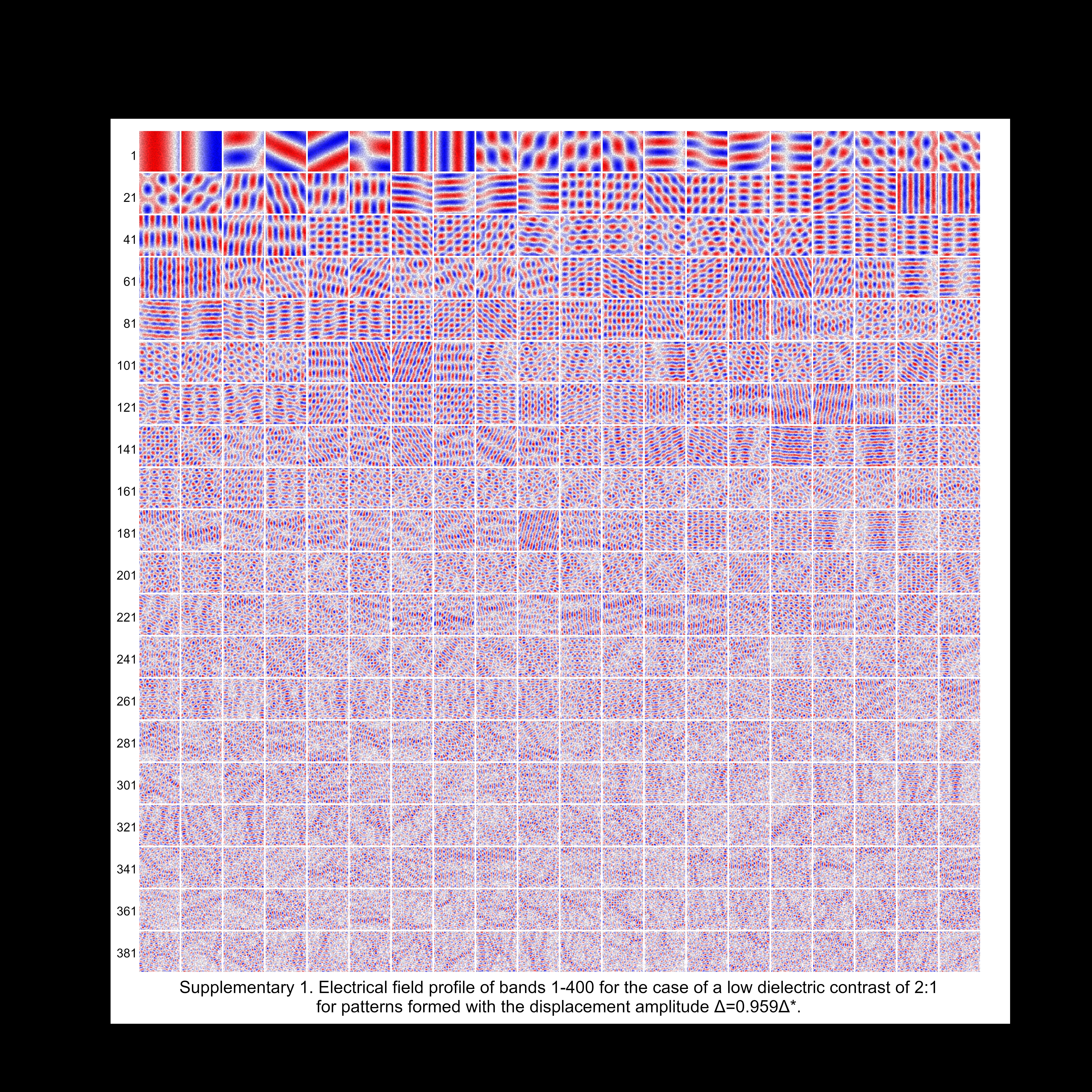

Supplement: Supplementary file 3 — Supplementary Material 3 [file 41598_2026_36235_MOESM3_ESM.jpg]

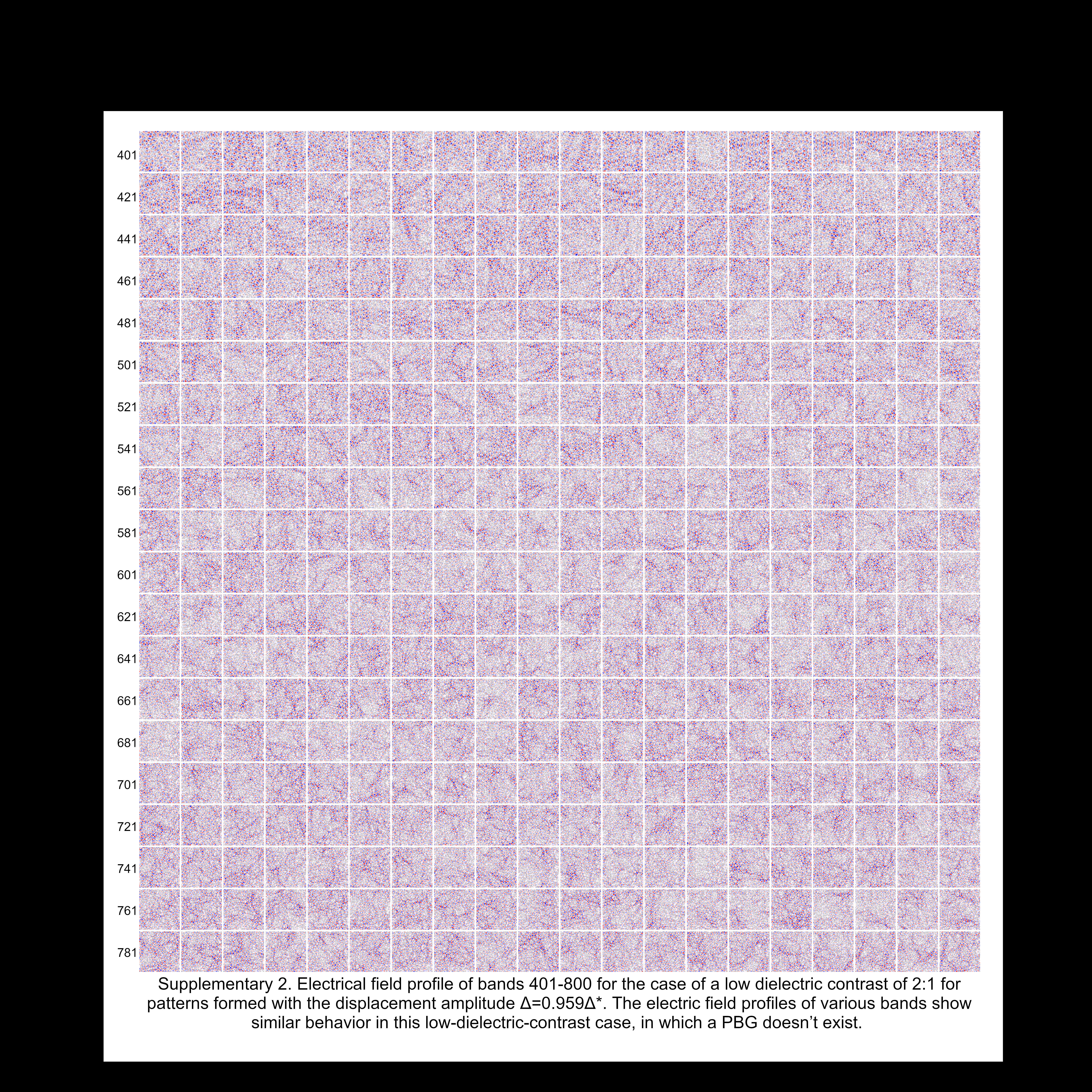

Supplement: Supplementary file 4 — Supplementary Material 4 [file 41598_2026_36235_MOESM4_ESM.jpg]
